# The Combinational Use of CRISPR/Cas9 and Targeted Toxin Technology Enables Efficient Isolation of Bi-Allelic Knockout Non-Human Mammalian Clones

**DOI:** 10.3390/ijms19041075

**Published:** 2018-04-04

**Authors:** Satoshi Watanabe, Takayuki Sakurai, Shingo Nakamura, Kazuchika Miyoshi, Masahiro Sato

**Affiliations:** 1 Animal Genome Research Unit, Division of Animal Science, National Institute of Agrobiological Sciences, Ibaraki 305-8602, Japan; 2 Basic Research Division for Next-Generation Disease Models and Fundamental Technology, Research Center for Next Generation Medicine, Shinshu University, Nagano 390-8621, Japan; tsakurai@shinshu-u.ac.jp; 3 Division of Biomedical Engineering, National Defense Medical College Research Institute, Saitama 359-8513, Japan; snaka@ndmc.ac.jp; 4 Laboratory of Animal Reproduction, Faculty of Agriculture, Kagoshima University, Kagoshima 890-8544, Japan; kmiyoshi@agri.kagoshima-u.ac.jp; 5 Section of Gene Expression Regulation, Frontier Science Research Center, Kagoshima University, Kagoshima 890-8544, Japan; masasato@m.kufm.kagoshima-u.ac.jp

**Keywords:** α-Gal epitope, α-1,3-galactosyltransferase, CRISPR/Cas9, endo-β-galactosidase C, targeted toxin, bi-allelic KO, indel mutations, isolectin BS-I-B_4_

## Abstract

Recent advances in genome editing systems such as clustered regularly interspaced short palindromic repeats/CRISPR-associated protein-9 nuclease (CRISPR/Cas9) have facilitated genomic modification in mammalian cells. However, most systems employ transient treatment with selective drugs such as puromycin to obtain the desired genome-edited cells, which often allows some untransfected cells to survive and decreases the efficiency of generating genome-edited cells. Here, we developed a novel targeted toxin-based drug-free selection system for the enrichment of genome-edited cells. Cells were transfected with three expression vectors, each of which carries a guide RNA (gRNA), humanized Cas9 (*hCas9*) gene, or *Clostridium perfringens*-derived endo-β-galactosidase C (*EndoGalC*) gene. Once EndoGalC is expressed in a cell, it digests the cell-surface α-Gal epitope, which is specifically recognized by BS-I-B_4_ lectin (IB4). Three days after transfection, these cells were treated with cytotoxin saporin-conjugated IB4 (IB4SAP) for 30 min at 37 °C prior to cultivation in a normal medium. Untransfected cells and those weakly expressing EndoGalC will die due to the internalization of saporin. Cells transiently expressing EndoGalC strongly survive, and some of these surviving clones are expected to be genome-edited bi-allelic knockout (KO) clones due to their strong co-expression of gRNA and *hCas9.* When porcine α-1,3-galactosyltransferase gene, which can synthesize the α-Gal epitope, was attempted to be knocked out, 16.7% and 36.7% of the surviving clones were bi-allelic and mono-allelic knockout (KO) cells, respectively, which was in contrast to the isolation of clones in the absence of IB4SAP treatment. Namely, 0% and 13.3% of the resulting clones were bi-allelic and mono-allelic KO cells, respectively. A similar tendency was seen when other target genes such as DiGeorge syndrome critical region gene 2 and transforming growth factor-β receptor type 1 gene were targeted to be knocked out. Our results indicate that a combination of the CRISPR/Cas9 system and targeted toxin technology using IB4SAP allows efficient enrichment of genome-edited clones, particularly bi-allelic KO clones.

## 1. Introduction

Gene targeting technology, which results in knockout (KO), is laborious and time-consuming. It comprises several steps that include the construction of a target vector for successful gene targeting, transfection of embryonic stem (ES) cells with the constructed vector, selection of transfectants with appropriate drugs, molecular biological screening of successfully targeted clones, blastocyst injection with the targeted ES clone to generate chimeric mice, acquiring heterozygous mice by mating with normal mice, and finally generating homozygous KO mice by mating between the resulting heterozygous mice. Thus, it typically takes 2 years or more to obtain homozygous mice from the transfection of the target vector. Furthermore, gene targeting is not applicable to larger animals such as pigs and bovines due to the lack of suitable germ-line competent ES cells in these animals. Instead, somatic cell nuclear transfer (SCNT) using embryonic fibroblastic cells as SCNT donors has been employed to generate KO animals. However, even in this case, it takes more than 2 years to obtain homozygous animals [1].

Recently, gene knockout has been successfully achieved through novel genome editing techniques—zinc-finger nuclease (ZFN), transcription activator-like effector nucleases (TALEN), and the clustered regularly interspaced short palindromic repeats (CRISPR)/CRISPR-associated protein-9 nuclease (Cas9) or CRISPR/Cas9 [2,3,4]. ZFN and TALEN cause mutations at the desired loci in the absence of donor DNA. They can introduce double-stranded breaks (DSBs) at the target site in the host chromosome, which are repaired by nonhomologous-end-joining (NHEJ). The NHEJ-based repair process generates the insertion or deletion of very few nucleotides, called “indel mutation (indel)”, which causes a frame-shift and disables encoded proteins or the formation of premature stop codons, and finally leads to the generation of a loss-of-function allele [5,6,7,8]. CRISPR/Cas9-based genome editing requires a synthetic guide RNA (gRNA) that can bind to the specific site of chromosomal DNA along with Cas9 [5,6,7,8]. The desired target sequence recognized by the gRNA/Cas9 complex must immediately precede a 5′-NGG protospacer adjacent motif (PAM). The CRISPR/Cas9 system has become widely used for efficient genome editing in various kinds of organisms, including non-human mammalian cells in rabbits [9], pigs [10,11,12], and bovines [13].

These genome-editing systems are noteworthy for generating bi-allelic KO cells in which the target gene is completely suppressed after single transfection with DNA or mRNA. However, the suppression of the target gene depends on the type of cells used, the type of genome editing systems employed, and the transfection efficiency [14]. CRISPR/Cas9-mediated genome editing is frequently employed for the targeted destruction of a specific locus. It involves co-transfection with gRNA and Cas9 expression vectors in which either an antibiotic resistance marker (i.e., puromycin *N*-acetyltransferase gene (*pac*)) or a fluorescent protein reporter (i.e., green fluorescent protein (GFP)) is included [15,16,17,18]. Cells transfected with genome editing reagents carrying a fluorescent protein reporter gene are subjected to fluorescence-activated cell sorting (FACS) 2–3 days after transfection. This approach is rapid and efficient for the enrichment of edited cells, but requires an expensive machine and efficient operational manpower. To enrich edited cells, using a Cas9 expression plasmid that carries an antibiotic resistance marker gene would be more convenient and cheaper. Generally, 2–4 days after transfection cells are incubated in a medium containing a selective drug (such as puromycin) for 3–5 days to remove untransfected cells (according to the “CRISPR KO Transfection Protocol” (https://www.scbt.com/protocols/CRISPR%20Protocol.pdf), Santa Cruz Biotechnology, Inc., Dallas, TX, USA). However, this transient expression system has several drawbacks. First, it can cause occasional chromosomal integration of Cas9 or gRNA, which may affect cell survival and function if it integrates into an essential locus for cell function. Second, transient treatment with these drugs allows some untransfected cells to survive, which may decrease the efficiency of generating genome-edited cells. To overcome these challenges, novel techniques without using selective drugs must be developed to enrich genome-edited cells.

**Figure 1:**
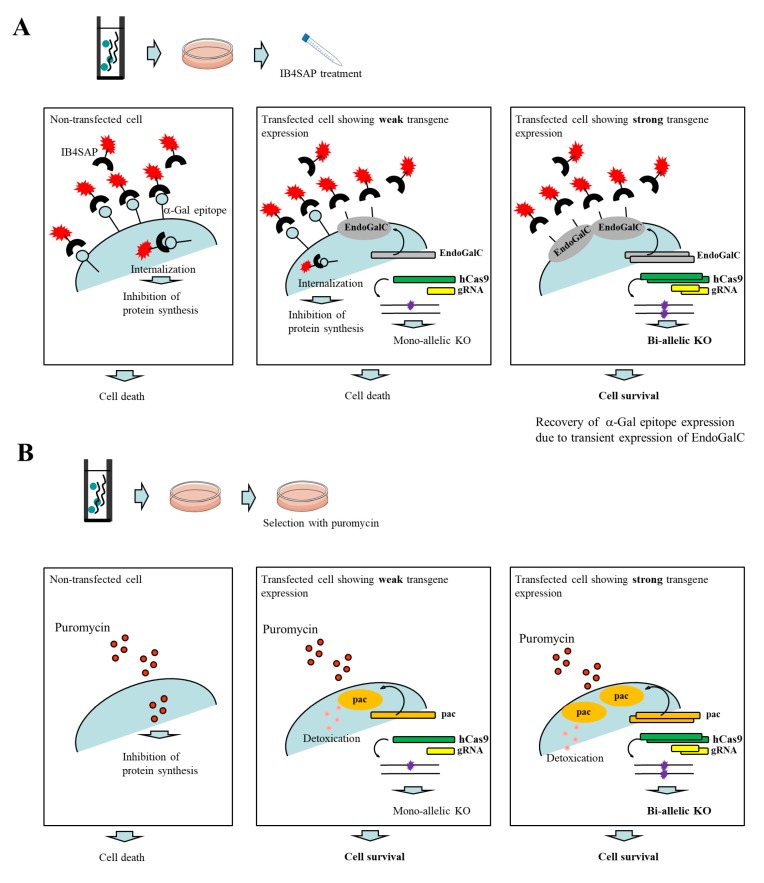
(**A**) Schematic representation of the isolation of genome-edited cells using EndoGalC-mediated digestion of the α-Gal epitope, CRISPR/Cas9-based genome editing, and IB4SAP-mediated targeted toxin technology. When cells are transfected with three expression vectors containing *EndoGalC*, *hCas9*, or gRNA targeted to a target locus, and 3 days later they are treated with IB4SAP for a short period, cells strongly expressing EndoGalC will survive and concomitantly both alleles of a target locus are expected to be knocked out (shown in the right panel). In contrast, cells weakly expressing EndoGalC and untransfected cells (all of which still express the α-Gal epitope on their surface), will undergo cell death (shown in the middle panel). (**B**) Schematic representation of the isolation of genome-edited cells using conventional puromycin-based genome editing. When cells are transfected with three expression vectors containing *pac*, *hCas9*, or gRNA targeted to a target locus, and 2~3 days later they are selected with puromycin for 2 days, the surviving clones are expected to be a mixture of those with mono-allelic knockout (KO) and bi-allelic KO phenotypes (shown in the middle and right panels).

Endo-β-galactosidase C (EndoGalC), derived from *Clostridium perfringens*, can digest the α-Gal epitope (Galα1-3Galβ1-4GlcNAc-R) expressed on the cell surface in non-human mammalian cells [19,20]. This epitope is easily recognized by staining cells with the α-Gal epitope-specific isolectin, BS-I-B_4_ (IB4) [21]. Therefore, cells transfected with an *EndoGalC* expression vector fail to react with IB4 [22]. Using this unique inverse-relationship between decreased reactivity to IB4 and increased EndoGalC expression, Sato et al. [23,24] developed a novel drug-free selection system for genetically modified cells. Based on the background of this study, we developed a novel CRISPR/Cas9-based approach to enrich genome-edited non-human cells having bi-allelic KO alleles in a drug-free condition. A schematic representation detailing this approach is shown in Figure 1A. An intact cell expresses the α-Gal epitope on its cell surface. When it is treated with cytotoxin saporin (SAP)-conjugated IB4 (hereinafter referred to as IB4SAP) for a short period (2 h, 37 °C), and then returned to the normal medium (without any selective drugs), IB4SAP binds to the α-Gal epitope and subsequently the complex is internalized by the cell. SAP (released from the internalized complex) inhibits protein synthesis, leading to cell death ([25]; left panel of Figure 1A). When cells are transfected with *EndoGalC*, humanized Cas9 (*hCas9*), and gRNA expression vectors, and subsequently treated with IB4SAP, only cells with complete loss of the α-Gal epitope (due to the transient overexpression of EndoGalC) survive, as they cannot bind to IB4SAP (right panel of Figure 1A). Hence, overexpressed gRNA and *hCas9* protein contribute to induce the efficient generation of bi-allelic KO mutations at the target locus (right panel of Figure 1A). Cells weakly expressing EndoGalC die, as they still bind to IB4SAP (middle panel of Figure 1A). Notably, these cells are expected to be genome-edited cells (probably having a mono-allelic mutation at the target locus) due to the weak expression of the simultaneously introduced CRISPR/Cas9 components (*hCas9* and gRNA expression vectors) (middle panel of Figure 1A). On the other hand, in the case of the puromycin-based selection of genome-edited cells, cells can survive in a medium containing puromycin when they are successfully transfected with a cocktail containing CRISPR/Cas9 components and *pac* expression plasmid. In this case, cells showing weak expression of transgenes can detoxicate the drug, but may exhibit a mono-allelic KO mutation due to the weak expression of CRISPR/Cas9 components (middle panel of Figure 1B). Cells showing strong expression of transgenes can detoxicate the drug, and would also frequently suffer from bi-allelic KO mutation induction as a result of the overexpression of genome-editing components (right panel of Figure 1B). Thus, the surviving clones are expected to be those that have a mixture of mono-allelic and bi-allelic KO mutations.

In this study, we performed proof of principle studies to show that CRISPR/Cas9 genome editing combined with the targeted toxin-based drug-free selection system is useful for the enrichment of non-human mammalian cells having bi-allelic KO mutations at the target locus using mouse ES cells, porcine embryonic fibroblasts (PEF), and porcine adipocyte precursor cells (PAPC).

## 2. Results

The aim of this study was to prove that cells surviving after co-transfection with CRISPR/Cas9-related DNA (gRNA and *hCas9* expression vectors) and an *EndoGalC* expression vector, as well as subsequent selection with IB4SAP, have a higher degree of indels leading to bi-allelic KO mutations in a target gene, in comparison to those obtained after transfection with CRISPR/Cas9-related DNA alone and no IB4SAP treatment or those obtained after transfection with CRISPR/Cas9-related DNA plus *pac*-containing plasmid DNA and subsequent selection in the presence of puromycin.

**Figure 2:**
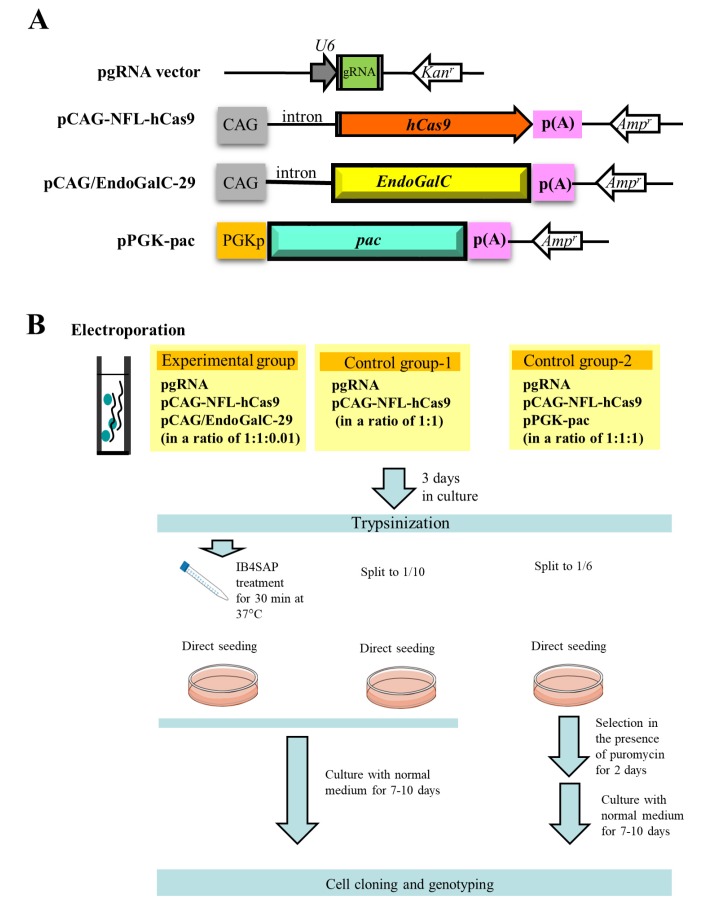
(**A**) Schematic representation of expression vectors, pgRNA vector, pCAG-NFL-*hCas9*, pCAG/*EndoGalC*-29, and pPGK-pac. CAG, cytomegalovirus enhancer + chicken β-actin promoter; hCas9, humanized Cas9 gene; p(A), poly(A) tail; *EndoGalC*, *Clostridium perfringens*-derived endo-β-galactosidase gene; *Kan^r^*, kanamycin resistance gene; *Amp^r^*, ampicillin resistance gene; U6, human *U6* promoter; PGKp, mouse phosphoglycerol kinase promoter; *pac*, puromycin *N*-acetyltransferase gene. (**B**) Flowchart of the experiments used to test the feasibility of a new system for the enrichment of genome-edited cells. Cells are co-transfected with three vectors, namely pgRNA, pCAG-NFL-hCas9, and pCAG/*EndoGalC*-29, as the experimental group. Three days after transfection, cells are treated with IB4SAP for a short period, prior to cultivation in a normal medium. For control group-1, cells are transfected with pgRNA and pCAG-NFL-*hCas9*. Three days after transfection, they are split to one tenth of the total amount, prior to cultivation in a normal medium. For control group-2, cells are transfected with pgRNA, pCAG-NFL-*hCas9*, and pPGK-*pac*. Three days after transfection, they are split to one sixth of the total amount, prior to cultivation in a medium containing puromycin for 2 days. The emerging colonies are propagated for molecular biological and cytochemical analyses.

The outlines of experiments are as shown in Figure 2B schematically. In the experimental group, three vectors (pgRNA#3 ([10]; Figure 2A) targeted to α-1,3-galactosyltransferases (*GAAT1*) gene (Table 1), pCAG-NFL-*hCas9* ([26]; Figure 2A), and pCAG/*EndoGalC*-29 ([22]; Figure 2A) at a ratio of 1:1:0.01) were transfected into PEF by electroporation. After transfection, cells were cultured in a normal medium for 3 days, trypsinized, and treated with IB4SAP for a short duration. The treated cells were again cultured in a normal medium until visible colonies were observed. In the control group-1, cells were similarly transfected in the presence of two vectors (pgRNA#3 and pCAG-NFL-*hCas9* at a ratio of 1:1), and cultured in normal medium for 3 days prior to trypsinization. One tenth of the cells were then cultured in a normal medium until visible colonies were observed. In the control group-2, three vectors (pgRNA#3, pCAG-NFL-*hCas9* and pPGK-pac ([27]; Figure 2A) at a ratio of 1:1:1) were transfected into PEF by electroporation. After transfection, cells were cultured in a normal medium for 3 days, trypsinized, and then one sixth of the cells were cultured in a medium containing puropmycin for 2 days. They were then cultured in a normal medium until visible colonies were observed.

**Figure 3:**
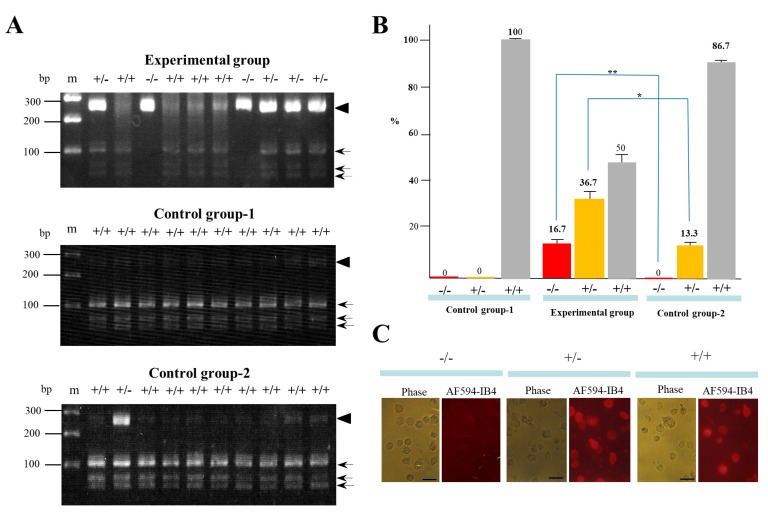
(**A**) An assay using the Guide-it™ Genotype Confirmation Kit of PCR products from clones after transfection of CRISPR/Cas9-related components targeting *GAAT1* locus + pCAG/*EndoGalC*-29 and subsequent IB4SAP treatment (experimental group), from those after transfection of CRISPR/Cas9-related components targeting *GAAT1* locus alone and no treatment with IB4SAP (control group-1), and from those after transfection of CRISPR/Cas9-related components targeting *GAAT1* locus + pPGK-pac (control group-2). The symbols (−/−, +/−, and +/+) above each lane indicate bi-allelic KO, mono-allelic KO, and wild-type cells, respectively. The arrowhead indicates the size of the parental PCR product that is still intact. Arrows indicate cleaved PCR products. M, 100 bp-ladder markers. (**B**) A summary of the genotyping of clones in each group was obtained after an assay using the Guide-it™ Genotype Confirmation Kit. Experiments were performed three times and all of the data were subjected to statistical analysis. The symbol “*” indicates no statistical significance (*p* > 0.5). The symbol “**” indicates statistical significance (*p* < 0.5). (**C**) Cytochemical staining of clones (judged as −/−, +/−, or +/+ by an assay using the Guide-it™ Genotype Confirmation Kit) with AF594-IB4. Note that cells judged as −/− were virtually negative for staining, while cells judged as +/− or +/+ were uniformly stained with the lectin. Phase, photographs taken under light microscopy; AF594-IB4, photographs taken under UV illumination to detect AF594-IB4–derived red fluorescence. Bar = 30 μm.

A total of 30 clones in each group (with three independent trials) were isolated and propagated for molecular biological and cytochemical analyses. To examine the rate of indel mutation induction among the isolated clones, we used a Guide-it™ Genotype Confirmation Kit (#Z2611N) provided by TaKaRa Bio, Inc. (Shiga, Japan). This kit provides a simple method to determine if a given clone has mutations in one allele (mono-allelic), both alleles (bi-allelic), or is unchanged (wild-type). As shown in the Materials and Methods section, this kit involves the amplification of the target site from cell clones obtained after transfection with a cocktail containing *hCas9* and gRNA expression plasmid DNA. The resulting amplicon is used in an in vitro cleavage reaction with recombinant hCas9 and the same gRNA used for the transfection experiment. The genotype can be determined by resolving the cleavage products on an agarose gel. A portion of the results of genome editing of *GAAT1* obtained using this assay is shown in Figure 3A. In the experimental group, the symbol (+/−) denoted on each lane was identified as a mono-allelic KO mutation, due to the presence of several bands of ~280 bp (parental products; denoted by the arrowhead in Figure 3A), ~100 bp, and <100 bp (cleaved products; denoted by arrows in Figure 3A). On the other hand, the symbol (−/−) denoted on each lane was identified as a bi-allelic KO mutation, due to the presence of a single band of ~280 bp alone. Samples in lanes denoted as +/+ had cleaved bands of 100 bp and <100 bp. Thus, 16.7% and 36.7% of the samples obtained from the experimental group were classified as −/− and +/−, respectively (Figure 3B). The remaining samples (50%) were identified as +/+ (Figure 3B). In the control group-1, which involved transfection with CRISPR/Cas9-related DNA alone and no IB4SAP treatment, all of the samples tested were identified as +/+ (middle panel of Figure 3A; Figure 3B). In the control group-2, which involved transfection with CRISPR/Cas9-related DNA plus *pac*-containing plasmid DNA and subsequent selection in the presence of puromycin, there was no −/− sample (lower panel of Figure 3A; Figure 3B). The rates of +/− and +/+ samples were respectively 13.3% and 86.7% (lower panel of Figure 3A; Figure 3B). Clones judged as −/−, +/−, or +/+ phenotypes by the above Guide-it™ Genotype Confirmation Kit were also confirmed by staining with Alexa Fluor 594 (AF594)-labeled IB4 (AF594-IB4) (Figure 3C). The cells judged as −/− exhibited no staining, while those judged as +/− or +/+ were positive for staining with the lectin. These results suggest the usefulness of IB4SAP for the enrichment of bi-allelic KO clones after transfection with CRISPR/Cas9-related DNA + *EndoGalC* expression vector. 

**Figure 4:**
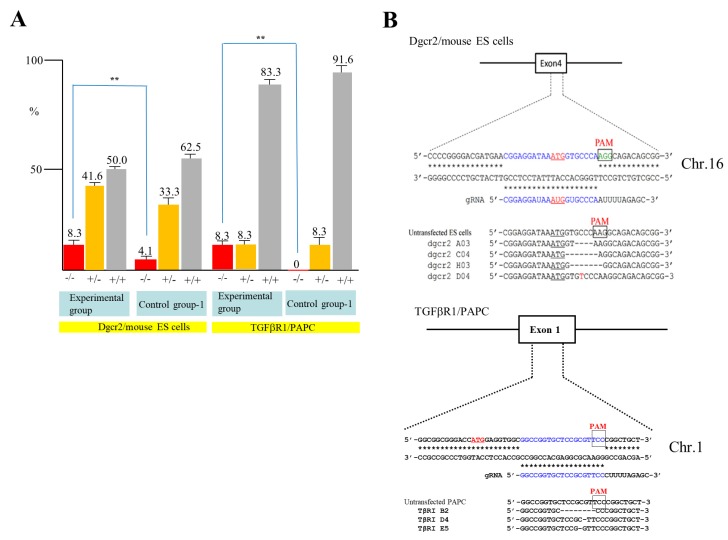
(**A**) A summary of the genotyping of clones from *Dgcr2* KO TT2 and *TGFβRI* KO PAPC obtained after an assay using the Guide-it™ Genotype Confirmation Kit. The symbol “**” indicates statistical significance (*p* < 0.5). (**B**) Sequencing of PCR products from *Dgcr2* KO TT2 (left panel) and *TGFβRI* KO PAPC (right panel). For *Dgcr2* KO TT2, three KO clones in the experimental group and untransfected ES cells (as control) were examined. ATG underlines indicate the translation initiation codon. Insertion of mononucleotide T in the clone dgcr2 D04 above the PAM is shown in red. The deleted portion in the clones dgcr2 A03, dgcr2 C04, and dgcr2 H03 is shown by dotted lines. Sequence from the mouse embryonic stem (ES) cells (as control) is also shown. For *TGFβRI* KO PAPC, three KO clones (TβRI B2, TβRI D4, and TβRI E5) in the experimental group and untransfected PAPC (as s control) are shown. Note that single nucleotide mutations were found in the TβRI D4 and TβRI E5 above the PAM sequence. The symbols −/−, +/−, and +/+ denoted above each lane indicate samples with bi-allelic KO, mono-allelic KO, and wild-type phenotypes, respectively.

To confirm the above results, we next attempted to destroy the DiGeorge syndrome critical region gene 2 (*Dgcr2*) and transforming growth factor-β receptor type 1 (*TGFβRI*) gene using mouse ES cells and PAPC. Mouse TT2 ES cells [30] were transfected with pgRNA#2 targeted to *Dgcr2* (Table 1), pCAG-NFL-*hCas9*, and pCAG/EndoGalC-29, and 3 days later they were subjected to IB4SAP, as shown in Figure 2B for the experimental group. Simultaneously, the control experiment (control group-1) was conducted by transfecting TT2 with pgRNA#2 and pCAG-NFL-*hCas9*, but no treatment with IB4SAP. A similar treatment was also performed in PAPC using pgRNA#4 targeted to *TGFβRI* (Table 1), pCAG-NFL-*hCas9*, and pCAG/*EndoGalC*-29 (experimental group) or pgRNA#4 and pCAG-NFL-*hCas9* (control group-1). A total of 24 clones in each group were isolated and propagated for molecular biological analysis. An assay using the Guide-it™ Genotype Confirmation Kit demonstrated that 8.3% of TT2 clones from the experimental group was judged as −/−, while 4.1% of clones from the control group-1 was judged as −/− (Figure 4A). Furthermore, 8.3% of PAPC cloned from the experimental group was judged as −/−, while there was no clone with the −/− genotype in the control group-1 (Figure 4A). Sequencing of the PCR products (sub-cloned into pPCR2.1) from randomly selected clones that had been identified as −/− or +/− revealed that all of the clones tested showed “indels”, in which a few nucleotides were deleted just above the PAM or there was the insertion of one nucleotide above the PAM (Figure 4B). Based on these results, we concluded that the combined use of the *EndoGalC* expression vector and IB4SAP-based selection system is effective for the enrichment of genome-edited cells, particularly bi-allelic KO clones.

## 3. Discussion

The novel genome editing system can isolate KO clones relatively faster and more effortlessly than the conventional selective gene targeting-based systems. However, isolating bi-allelic KO clones using the new systems can be laborious and time-consuming for researchers, because they must carefully analyze each isolated clone for possible mutations in the target locus by using several molecular biological tools such as the Surveyor assay (or T7 Endonuclease (T7E1) assay), sequencing, and Western blotting [2,3,4]. Therefore, the development of an effective method to enrich genome-edited clones, particularly bi-allelic KO clones, is necessary to overcome these challenges. To date, co-transfection using Cas9 expression vector, which carries either the fluorescent protein gene (i.e., *GFP*) or antibiotic resistance gene (i.e., *pac*), together with a vector for expressing a synthetic gRNA has been conventionally employed [15,16,17,18]. Transient expression of these drug resistance genes enables the isolation of genome-edited clones, but the target gene may not be fully edited in some of the surviving clones after drug selection, since even a slight expression of a drug resistance gene allows cells to confer resistance against selective drugs, which may occasionally generate mono-allelic KO clones together with bi-allelic ones, as shown in the middle panel of Figure 1B. Therefore, the enrichment of bi-allelic KO clones is needed to select only cells exhibiting overexpression of the foreign DNA including genome-editing components. In this sense, a targeted toxin-based cell selection system using IB4SAP is ideal for the enrichment of transgene-high expressing clones [22,23]. A short treatment with IB4SAP (0.5~2 h) eliminates α-Gal epitope-expressing untransfected cells and cells expressing EndoGalC weakly, as a result of the internalization of saporin (see left and middle panels of Figure 1A). Thus, cells surviving after IB4SAP treatment must be those exhibiting strong expression of EndoGalC (as well as CRISPR/Cas9-related components), although its expression was transient. Notably, Sato et al. [23,24] demonstrated that high expression levels of EndoGalC (evaluated by the loss of the α-Gal epitope) are closely associated with those of GFP from the co-transfected expression vector. From these present studies, our targeted toxin-based cell selection system was found to be more effective for the enrichment of bi-allelic KO clones than the conventional puromycin-based cell selection system.

In this study, to prove that our novel bi-allelic KO clone’s enrichment system is functional, we first aimed to destroy *GAAT1*, which is involved in synthesis of the α-Gal epitope, since loss of α-Gal epitope expression is easily monitored by cytochemical staining using AF594-IB4 [22]. In this case, an attempt to destroy the function of *GAAT1* might have also contributed to increase the cell survival rate after the transfection of genome-editing components and *EndoGalC* vector and the subsequent selection of α-Gal epitope-negative cells using IB4SAP. However, the puromycin-based selection of genome-edited PEF failed to produce bi-allelic KO clones (see “control group-2” in Figure 3B), which had been performed at the same time as the experimental group. Furthermore, our targeted toxin-based selection system proved useful for other target genes such as *Dgcr2* (this study), *TGFβRI* (this study), phosphatase and tensin homolog from chromosome 10 (*PTEN*) [31], *p53* [31], and a low-density lipoprotein receptor gene (*LDLR*) [31] using different non-human cells. Taking these into account, it would be reasonable to consider that our system is indeed useful for the enrichment of clones with bi-allelic KO mutations. 

Bi-allelic deletions were easily generated via the single transfection of a somatic cell with CRISPR/Cas9 systems targeting genes of interest. The resultant cells should exhibit complete loss of a protein encoded by the target gene, which is clearly in contrast to cells transfected with the knockdown vectors that confer RNA interference-based silencing, and will be used for analysis of the pathogenesis of genetic disease such as the recessive X-linked form of muscular dystrophy, the development of pharmaceuticals [32,33], and the creation of cloned genetically modified domestic animals that are derived from the SCNT of bi-allelic KO cells as SCNT donors [12,34]. Recently, for investigating epigenetic modifiers, the usefulness of bi-allelic null mutant mouse ES cells has been suggested [35].

To date, several methods for the enrichment of bi-allelic KO clones have been described. For example, Gundry et al. [36] demonstrated that electroporation-based introduction of hCas9–gRNA ribonucleoprotein complexes (*RNPs*) into wild-type cells resulted in highly efficient genome editing in murine and human hematopoietic progenitor cells. Takayama et al. [37] demonstrated that the overexpression of RAD51, a protein functioning as a facilitator of homologous recombination (HR) in the NHEJ-based repair pathway, and the treatment of valproic acid (VPA), a reagent involving in loosened nucleosome folding in transcriptionally inactive chromatin, enhanced bi-allelic targeting efficiency in human ES/iPS cells, especially for the transcriptional inactive targeted locus. In this context, the combinational use of *RNPs* or the overexpression of RAD51 and treatment of VPA with our targeted toxin-based selection system would enhance the acquisition of bi-allelic KO clones.

Our present system, based on the IB4SAP-mediated selection of α-Gal epitope-expressing cells, is limited to mammalian cells that express the α-Gal epitope, except for cells from humans and Old World monkeys. However, it may be a useful tool for genetically modified livestock produced by SCNT [38], which requires a genetically pure cell population (such bi-allelic KO cells) as SCNT donors.

## 4. Materials and Methods

### 4.1. Plasmid Vectors

The plasmid structure used in this study is schematically illustrated in Figure 2A. pgRNA vector (Addgene, Cambridge, MA, USA) has a human *U6* promoter to drive transcription of the downstream gRNA. The site where synthesized oligonucleotides (oligos) are inserted is colored green (Figure 2A). Double-stranded (ds) oligos used for insertion below the *U6* promoter in pgRNA vector were generated according to the manufacturer’s instructions. The sequences of oligos targeting to the specific endogenous gene are listed in Table 1. The plasmid pCAG-NFL-*hCas9* was used for hCas9 expression, which is composed of chicken β-actin-based promoter CAG [39], *hCas9* gene (carrying FLAG (NFL) at its 5′ end), and poly(A) sites from the rabbit β-globin gene. The *EndoGalC* expression vector, pCAG/*EndoGalC*-29, which has CAG promoter, *EndoGalC* gene, and poly(A) sites from the rabbit β-globin gene, was used. The *pac* expression vector, pPGK-pac, is comprised of mouse phosphoglycerate kinase (PGK) promoter, *pac*, and the poly(A) site from the mouse PGK gene. 

### 4.2. Cell Culture and Transfection

A TT2 mouse ES cell line was cultured in N2B27 serum-free medium containing 3 μM CHIR99021 (#1386; Axon, Groningen, The Netherlands), 0.5 μM PD184352 (#161-23701; Wako Pure Chemical Industries, Ltd., Tokyo, Japan), 1 μM SU5402 (#191-15271; Wako Pure Chemical Industries, Ltd.), and 1 × 10^3^ U/mL leukemia inhibitory factor (LIF; ESGRO; #ESG1107; Chemicon, Temecula, CA, USA) without s feeder cell layer at 37 °C in an atmosphere of 5% CO_2_ in air. PEF [40] and PAPC [41] were cultured in Dulbecco’s modified Eagle’s medium (DMEM; Sigma-Aldrich Co. Ltd., St. Louis, MO, USA) supplemented with 10% fetal bovine serum (FBS), 50 units of penicillin, and 50 µg/mL of streptomycin at 37 °C in an atmosphere of 5% CO_2_ in air.

For transfection, *hCas9*, gRNA, and *EndoGalC* expression vectors were mixed together in a ratio of pCAG-NFL-*hCas9*:pgRNA:pCAG/*EndoGalC*-29 = 1 (6 μg):1 (6 μg):0.01 (0.06 μg) as the experimental group. In the control experiment (control group-1), the pCAG/*EndoGalC*-29 vector was not used for transfection. Also, as another control experiment (control group-2), *hCas9*, gRNA, and *pac* expression vectors were mixed together in a ratio of pCAG-NFL-*hCas9*:pgRNA:pPGK-pac = 1 (6 μg):1 (6 μg):1 (6 μg). These vectors were introduced into the cells (10^7^ for TT2 and 10^6^ for both PEF and PAPC) by nucleofection-based electroporation (Lonza GmbH, Köln, Germany), according to the method described previously [42]. After gene transfer, TT2 cells were seeded onto 3 × 100-mm tissue culture dishes (#3020-100; Iwaki Co., Ltd., Tokyo, Japan), while PEF and PAPC were seeded onto 2 × 100-mm dishes. The seeded cells were cultured in a normal medium without any antibiotics.

In the case of genome editing of the *GAAT1* locus using PEF, electroporation-based gene delivery was performed three times for each group, and from each group 10 clones were isolated for genomic DNA analysis. Thus, a total of 30 clones were analyzed for each group. In the case of genome editing of the *Dgcr2* and *TGFβRI* loci using TT2 and PAPC, electroporation-based gene delivery was performed three times for the experimental group and control group-1, and from each group eight clones were isolated for genomic DNA analysis. Thus, a total of 24 clones were analyzed for each group.

### 4.3. IB4SAP Treatment

Cells were trypsinized 3 days after transfection and approximately 80% were incubated for 30 min at 37 °C in a solution (25 μL) containing 0.5–1.0 μg IB4SAP (#IT-10; Advanced Targeting Systems, Inc., San Diego, CA, USA), 5% FBS, and 1 mM CaCl_2_ in Dulbecco’s modified phosphate buffered saline without Ca^2+^ and Mg^2+^ (D-PBS), according to the method described previously [22]. The treated cells were directly seeded onto a 100-mm dish with normal culture medium and cultured for an additional 7–10 days. In the control (without IB4SAP treatment), a small number of cells (one sixth or one tenth of the trypsinized cells) were plated in a 100-mm dish and cultured in a normal medium for 7–10 days, until colonies were observed. The colonies were then picked up by a paper method [40] and cultured in 96-well plates (Iwaki Co. Ltd.) containing a normal medium, prior to step-wise propagation.

### 4.4. PCR and Sanger Sequencing of Mutated Sites

Genomic DNA was extracted by adding 100 μL of lysis buffer (0.125 μg/mL of proteinase K, 0.125 μg/mL of Pronase E, 0.32 M sucrose, 10 mM Tris-HCl (pH 7.5), 5 mM MgCl_2_, and 1% (*v*/*v*) Triton X-100) to the cell pellets (ranging from 10^3^ to 10^4^ cells/mL), and lysed with vigorous shaking overnight at 37 °C and extraction with phenol/chloroform [42]. The supernatant was isopropanol-precipitated, and the precipitated DNA was then dissolved in 20 μL of sterile water. The DNA was stored at 4 °C.

The resulting DNA samples (1 μL; approximately 5 ng) were subjected to the first PCR using primer sets (S1/AS1 series) for each target gene (Table S1) in a volume of 20 μL, using the PCR conditions previously described [42]. As controls, genomic DNA (~5 ng) isolated from intact TT2, PEF, and PAPC were concomitantly PCR-amplified. The expected band sizes of the resulting PCR products are shown in Table S1. In some cases, a second round of PCR (nested PCR) was performed using 1 μL of the first PCR product as a template. The PCR conditions used were the same as those employed for the first PCR, except for the primer sets (S2/AS2 series; Table S1). These resulting products (1 μL) were checked by loading on a 2% agarose gel. The remaining products were ethanol-precipitated, re-suspended in ~20 μL of sterile water, and their DNA content was measured using a spectrophotometer.

For sequencing, some of the PCR products were sub-cloned into a TA cloning vector pCR2.1 (Invitrogen Co., Carlsbad, CA, USA). Dye termination cycle sequencing was performed at FASMAC Co., Ltd. (Atsugi, Kanagawa, Japan).

### 4.5. Genotyping of Genome-Edited Cells

For the genotyping of genome-edited clones, we used the Guide-it™ Genotype Confirmation Kit. The procedure was followed according to the manufacturer’s instruction. The protocol involves PCR-based amplification of the target site and in vitro cleavage with Cas9 and the gRNA used for the original CRISPR/Cas9 gene editing experiment. If indels are present at the target site, the original gRNA/Cas9 complex will be unable to cleave the site, whereas wild-type alleles will be recognized and cleaved. Briefly, in vitro transcription of gRNAs was first conducted using the Guide-it sgRNA In Vitro Transcription Kit (#632635; TaKaRa Bio, Inc.). Furthermore, 1 µL of target-specific gRNA (50 ng/µL) and 0.5 µL of Guide-it Recombinant Cas9 Nuclease (500 ng/µL) were mixed in a 200-µL PCR tube to create a Cas9/gRNA mix, and then incubated using a thermal cycler with the following conditions: 37 °C for 5 min, and then cooling to 4 °C. Then, the PCR products (200–300 ng) generated from the amplification of genomic DNA isolated from each clone were mixed with Cas9 reaction buffer, bovine serum albumin, RNase-free water, and Cas9/gRNA mix in a total volume of 20 µL. The resulting products (10 µL) were then electrophoresed in a 2% agarose gel.

### 4.6. Staining with AF594-IB4 and Detection of Fluorescence

The staining of embryos with AF594-IB4 (#I21413; Invitrogen Co.) was conducted as described in our previous study [23]. The stained embryos were examined under a fluorescence microscope (BX60; Olympus, Tokyo, Japan) using DM505 (BP460-490 and BA510IF; Olympus, Tokyo, Japan) and DM600 filters (BP545-580 and BA6101F; Olympus), which were used to detect EGFP-derived green fluorescence and AF594-derived red fluorescence, respectively. Micrographs were taken using a digital camera (FUJIX HC-300/OL; Fujifilm Co., Tokyo, Japan) attached to the fluorescence microscope and images were printed using a Mitsubishi digital color printer (CP700DSA; Mitsubishi, Tokyo, Japan).

### 4.7. Statistics

Data are presented as the mean ± standard error. All percentage data were subjected to arcsine transformation for each replicate. The rates of clones having indels in each treatment group were analyzed using one-way ANOVA followed by Fisher’s protected least significant difference test. A probability of *p* < 0.05 was considered statistically significant.

## 5. Conclusions

We developed a novel system for the enrichment of genome-edited cells by the co-transfection of CRISPR-Cas9-related nucleic acids and an *EndoGalC* expression vector, and subsequent treatment with IB4SAP. Using this system, we successfully obtained bi-allelic KO cells from mouse ES cells, PEF, and PAPC with relatively good efficiency.

## Figures and Tables

**Table 1:** List of gRNA sequences in pgRNA vectors.

ID	Sequences (5′–3′) ^1^	Location	GenBank#	Reference
pgRNA#2	GGAGGATAAATGGTGCCCAAGG	*Dgcr2* exon4	BC062978.1	Kajiwara et al. [28]
pgRNA#3	AGAAAATAATGAATGTCAAAGG	*GAAT1* exon 4	NM0090657	Sato et al. [10]
pgRNA#4	GGCCGGTGCTCCGCGTTCCCGG	*TGFβRI* exon 1	NM_001038639	Vellucci et al. [29]

^1^ Protospacer adjacent motif (PAM) sequences are underlined.

## Supplementary Materials

The following are available online at http://www.mdpi.com/1422-0067/19/4/1075/s1.

## Abbreviations

AF594-IB4	Alexa Fluor 594-labeled BS-I-B_4_ isolectin
α-GalT	α-1,3-galactosyltransferase
CAG	Chicken β-actin-based promoter
CRISPR/Cas9	Clustered regularly interspaced short palindromic repeats (CRISPR)/CRISPR-associated protein-9 nuclease
D-PBS	Dulbecco’s modified phosphate-buffered saline without Ca^2+^ and Mg^2+^, pH 7.4
ds	Double-stranded
DSBs	Double-stranded breaks
GFP	Green fluorescent protein
EndoGalC	Endo-β-galactosidase C
ES	Embryonic stem
FACS	Fluorescence-activated cell sorting
FBS	Fetal bovine serum
*Dgcr2*	DiGeorge syndrome critical region gene 2
DMEM	Dulbecco’s modified Eagle’s medium
FBS	Fetal bovine serum
*GAAT1*	α-1,3-galactosyltransferase
gRNA	Guide RNA
GFP	Green fluorescent protein
hCas9	Humanized Cas9
HR	Homologous recombination
IB4SAP	Saporin toxin-labeled BS-I-B_4_ isolectin
iPS	Induced pluripotent stem
indels	Indel mutations
KO	Knockout
LDLR	Low density lipoprotein receptor
NHEJ	Nonhomologous-end-joining
oligos	Oligonucleotides
*pac*	Puromycin *N*-acetyltransferase gene
PAM	Protospacer adjacent motif
PAPC	Porcine adipocyte precursor cells
PGK	Phosphoglycerate kinase
PEF	Porcine embryonic fibroblasts
PCR	Polymerase chain reaction
PFA	Paraformaldehyde
PTEN	Phosphatase and tensin homolog from chromosome 10
RNP	Ribonucleoprotein complexes
SAP	Saporin
SCNT	Somatic cell nuclear transfer
T7E1	T7 Endonuclease assay
TALEN	Transcription activator-like effector nucleases
*TGFβRI*	Transforming growth factor-β receptor type 1 gene
VPA	Valproic acid
ZFN	Zinc-finger nuclease
