# Supplementary material for: The Combinational Use of CRISPR/Cas9 and Targeted Toxin Technology Enables Efficient Isolation of Bi-Allelic Knockout Non-Human Mammalian Clones
Source: Int J Mol Sci. 2018 Apr 4;19(4):1075. doi: 10.3390/ijms19041075 (PMC5979347; doi:10.3390/ijms19041075)
Supplement: Supplementary file 1 [file ijms-19-01075-s001.pdf]

Supplementary

The Combinational Use of CRISPR/Cas9 and Targeted Toxin Technology Enables Efficient Isolation of Bi-Allelic Knockout Non-Human Mammalian Clones

Satoshi Watanabe [1,*], Takayuki Sakurai [2], Shingo Nakamura [3], Kazuchika Miyoshi [4] and Masahiro Sato [5]

1 Animal Genome Research Unit, Division of Animal Science, National Institute of Agrobiological Sciences, Ibaraki 305-8602, Japan
2 Basic Research Division for Next-Generation Disease Models and Fundamental Technology, Research Center for Next Generation Medicine, Shinshu University, Nagano 390-8621, Japan; tsakurai@shinshu-u.ac.jp
3 Division of Biomedical Engineering, National Defense Medical College Research Institute, Saitama 359-8513, Japan; snaka@ndmc.ac.jp
4 Laboratory of Animal Reproduction, Faculty of Agriculture, Kagoshima University, Kagoshima 890-8544, Japan; kmiyoshi@agri.kagoshima-u.ac.jp
5 Section of Gene Expression Regulation, Frontier Science Research Center, Kagoshima University, Kagoshima 890-8544, Japan; masasato@m.kufm.kagoshima-u.ac.jp
* Correspondence: kettle@affrc.go.jp; Tel.: +81-29-838-8662

Table S1: Primer sets used for PCR.

Target Gene	Primer	Sequence (5′-3′)	Reference	Expected size of generated band
Mouse *Dgcr2*	S1	GGGGTCACGCGGCTGCCCCG	Kajiwara *et al.* [1]	100 bp
	AS1	ACAGTGAGAACCAGCAGGAA		60 bp
	S2	GGGACGATGAACGGAGGATA		
	AS2	GAGCAGCAGGAAGGCACCGC		
Porcine *GAAT1*	S1	GCAAATTAAGGTAGAACGCA	Sato *et al.* [2]	230 bp
	AS1	TTCCCAAAACACAACCATTA		150 bp
	S2	AGAAAAGATATTGGTATAAG		
	AS2	CAGTTGAGACAAGCAGCATT		
Porcine *TGFβRI*	S1	GGCGGGACCTGGAGGTGGC	Vellucci *et al.* [3]	62 bp
	AS1	CAGCACGAAGAGGAGCAGCC		91 bp
	S2	ATGGAGGTGGCGGCCGGTGC		
	AS2	TGGAATGCCGTCGGCTCCGG		

1. Kajiwara, K.; Nagasawa, H.; Shimizu-Nishikawa, K.; Ookura, T.; Kimura, M.; Sugaya, E. Cloning of SEZ-12 encoding seizure-related and membrane-bound adhesion protein. *Biochem. Biophys. Res. Commun.* **1996**, *222*, 144–148.
2. Sato, M.; Miyoshi, K.; Nagao, Y.; Nishi, Y.; Ohtsuka, M.; Nakamura, S.; Sakurai, T.; Watanabe, S. The combinational use of CRISPR/Cas9-based gene editing and targeted toxin technology enables efficient biallelic knockout of the alpha-1,3-galactosyltransferase gene in porcine embryonic fibroblasts. *Xenotransplantation* **2014**, *21*, 291–300.
3. Vellucci, V.F.; Reiss, M. Cloning and genomic organization of the human transforming growth factor-beta type I receptor gene. *Genomics* **1997**, *46*, 278–283.
